# Mobilizing civil society for the HIV treatment cascade: a global analysis on democracy and its association with people living with HIV who know their status

**DOI:** 10.1002/jia2.25374

**Published:** 2019-08-04

**Authors:** Rayner KJ Tan, Chen Seong Wong

**Affiliations:** ^1^ Saw Swee Hock School of Public Health National University of Singapore Singapore; ^2^ National Centre for Infectious Diseases Singapore; ^3^ Department of Medicine Yong Loo Lin School of Medicine National University of Singapore Singapore

**Keywords:** Democracy index, HIV criminalization, HIV cascade, testing, civil society, community mobilization

## Abstract

**Introduction:**

Civil society organizations (CSOs) play an essential role in the global HIV/AIDS response. Past studies have described the beneficial role of CSOs in meeting the United Nations Programme on HIV/AIDS (UNAIDS) 90‐90‐90 target, but have not explored how political conditions, which influence the ability of CSOs to organize, have an impact on the cascade. This study explores the relationship between measures of democracy and its association with diagnosis rates among people living with HIV (PLHIV).

**Methods:**

This study analyses 2016 data derived from the Economist Intelligence Unit's Democracy Index (EIUDI), UNAIDS country estimates for PLHIV and PLHIV who knew their status in 2016, World Bank's 2016 data on nominal gross domestic product (GDP) per capita and country population, HIV Justice Network's 2016 data on HIV criminalization, and country‐level estimates for PLHIV, PLHIV who know their status, and expenditure on HIV prevention from other independent sources. An estimated HIV prevalence variable was constructed by dividing the estimated PLHIV population with the total population of a country. Analyses were limited to countries with available data on PLHIV who know their status (n = 111).

**Results:**

Of the 111 countries in the analytic sample, the mean democracy index score was 5.93 (of the 10), median estimated HIV prevalence was 0.20% (IQR 0.10‐0.65), median GDP per capita (in thousands, US dollar) was 4.88 (IQR 2.11‐13.79), and mean PLHIV who know their status is 67.12%. Preliminary analysis on the five component measures of the EIUDI revealed multicollinearity, and thus the composite democracy index score was used as the measure for democracy. Multivariate linear regression analyses revealed that democracy index scores (β = 2.10, SE = 1.02, *p* = 0.04) and GDP per capita (in thousands; β = 0.34. SE = 0.11, *p* < 0.01) were positively associated with diagnosis rates among PLHIV, controlling for country‐level expenditure on HIV prevention, HIV criminalization laws and estimated HIV prevalence.

**Conclusions:**

Results indicate that higher levels of democracy were positively associated with rates of diagnosis among PLHIV. Further analyses following wider implementation of universal testing and treatment is warranted, as well as the need for further research on the mechanisms through which political cultures specifically influence rates of diagnosis among PLHIV.

## 1 Introduction

A total of approximately 36.9 million people were living with HIV (PLHIV) as of 2017, of whom 9.4 million were estimated to not have known that they were living with HIV 1. The “90‐90‐90” treatment cascade targets were introduced in 2014 by the Joint United Nations Programme on HIV/AIDS (UNAIDS); the goal was to ensure that by the year 2020, 90% of all PLHIV will know their status, 90% of all people who have been diagnosed with HIV will be on effective antiretroviral therapy (ART), and 90% of all people who are on ART will have viral suppression 2. In 2017, the world had achieved 75‐79‐81, with regions in the Sub‐Saharan Africa reporting lower percentages on the treatment cascade, relative to rest of the world 3.

Civil society organizations (CSOs) have been at the forefront of the HIV/AIDS response, and play an essential role in the global fight against HIV/AIDS, especially by serving as a bridge between policymakers and members of communities that are affected disproportionately by HIV/AIDS. These organizations typically serve as “gatekeepers” and possess expertise and knowledge of the individuals in the communities that they serve 4. Past studies have shown that community mobilization and CSO engagement in communities was associated with greater knowledge of HIV/AIDS, changes in attitudes towards HIV risk behaviours, access to HIV/AIDS‐related services, and greater health‐seeking behaviours that include HIV testing, and the uptake of HIV prevention methods among community members 5, 6, 7, 8, 9, 10.

In spite of the focus on the role of CSOs, less attention in the extant literature has focused on the pre‐conditions that allow CSOs to thrive as institutions of healthcare provision for HIV prevention. In general, past studies have studied the link between regime type and health, and found a positive relationship between measures of democracy and health outcomes such as increased life expectancy and reduced infant mortality 11, 12. In the context of HIV, studies have shown how greater state capacity is positively associated with lower HIV infection rates 13, and how democratic conditions increase access to HIV prevention and treatment 14, 15. Scholars have attributed these findings to how democratic political conditions enable active non‐governmental and citizen participation in healthcare, while according them greater civil liberties 16, which hold true as in the context of HIV organizational leadership 17, 18. Nevertheless, several scholars find that regardless of regime type, contextual and historical precedence may impede or facilitate such responses to the epidemic 19, 20.

In light of the scholarly work that draws attention to how regime types and democratic political conditions serve to improve health outcome through citizen engagement in healthcare, we propose that this relationship remains salient in the sphere of HIV prevention, and particularly, for the diagnosis of PLHIV. This study attempts to extend existing work in the field and make a contribution through the lens of the UNAIDS 90‐90‐90 targets by conducting a global analysis on country‐level measures of democracy and its association with the first “90” of the HIV treatment cascade; PLHIV who know their HIV status.

## 2 Methods

The dataset for this study was constructed based on available country‐level data on from several sources. The primary outcome, estimated percentage of PLHIV who know their status, was obtained from country estimates for PLHIV who knew their status in the year 2016 that were made available in the UNAIDS Data 2017 report, and is expressed as a continuous variable 3. As data was only available for 72 countries from this source, additional searches for published, online sources, including peer‐reviewed journal articles, country‐specific reports and other published data were undertaken to generate data points for an additional 39 countries 21, 22, 23, 24, 25, 26, 27, 28, 29.

The Economist Intelligence Unit's Democracy Index (EIUDI; or henceforth, democracy index) was employed as the study's main measure of democracy. The democracy index is a composite score that is calculated as the mean of five component scores, namely “electoral process and pluralism,” “civil liberties,” “functioning of government,” “political participation” and “political culture.” Each component is measured on a continuous scale and scored on a scale of 0 to 10. The democracy index is scored on a continuous scale of 0 to 10, with a higher number indicating higher levels of democracy within the country. While the Freedom in the World index by Freedom House and the Polity IV datasets have been widely used in academic research for similar research, we opted for the EIUDI as our outcome measure for its comparatively greater focus on the quality of democracy, especially in the areas of political freedoms and civil liberties, in its conceptualization of democracy 30, 31. Preliminary analysis utilizing the five component measures of the democracy index as independent variables revealed issues of multicollinearity, and thus the composite democracy index score was ultimately used as the measure for democracy in this study.

We collected data on other variables that could potentially confound the relationship between democracy and the proportion of PLHIV who know their status. We compiled 2016 data on country‐level nominal gross domestic product (GDP) per capita (current US$) through the World Bank 32. For countries without 2016 data available from this source, the data point for the last available year was used. Additionally, we computed the estimated HIV prevalence, in percentage, for each country by dividing the 2016 estimates for total number of PLHIV by the total population of the country, which were obtained from the UNAIDS database and World Bank respectively 33, 34. Total expenditure on HIV prevention programmes was obtained from a study conducted by the Global Burden of Disease Health Financing Collaborator Network that provided estimates for health spending in the year 2015, which was collected as a continuous variable, in US dollar (USD) millions 35. Lastly, data on the prevalence of HIV criminalization laws were obtained from the HIV Justice Network, and was collected as a binary, categorical variable (yes vs. no) 36; these include HIV‐specific criminals laws that specifically penalize PLHIV who know their status and who intentionally or unintentionally expose others to HIV, or general criminal laws (e.g. assault, reckless endangerment) that allow for the prosecution of PLHIV under these laws for acts such as potential or unintended exposure to HIV, or non‐disclosure of HIV status.

This study employed descriptive statistics to elucidate patterns and trends in country characteristics based on the constructed dataset. We also employed multivariate linear regression models to determine the relationship between the stated independent variables and the primary outcome variable, percentage of PLHIV who know their status. Multivariate analyses were limited to countries where data on PLHIV who know their status was available (n = 111). Quantitative data analysis was carried out using the statistical software STATA version 15 (Stata Corp, College Station, TX, USA). Statistical significance was set at *p* < 0.05.

## 3 Results and discussion

Table 1 summarizes the country‐level data that was compiled for this study. The mean democracy index score was 5.93 (of the 10), median estimated HIV prevalence was 0.20% (IQR 0.10‐0.65), median GDP per capita (in thousands, USD) was 4.88 (IQR 2.11‐13.79), median healthcare spending on HIV prevention (in millions, USD) was 23.91, and mean PLHIV who know their status is 67.1%. A total of 47 of the 111 countries (57.7%) had HIV criminalization laws.

**Table 1:** Summary of countries in analytic sample and country‐level data

Country	PLHIV who know their status (%)	Democracy index	Nominal GDP per capita (current USD)	Expenditure on HIV prevention (USD millions)	Criminalization of HIV	Estimated HIV prevalence (%)
Afghanistan	29.0	2.55	561.78	13.55	No	0.022
Albania	47.0	5.91	4124.98	1.93	Yes	0.059
Algeria	76.0	3.56	3916.88	6.60	No	0.032
Angola	40.0	3.40	3308.70	20.16	Yes	0.972
Argentina	79.0^a^	6.96	12,440.32	28.50	No	0.274
Armenia	60.0	3.88	3614.69	3.25	Yes	0.113
Australia	92.0^a^	9.01	49,927.82	64.61	No	0.104
Austria	88.0^a^	8.41	44,676.35	30.67	No	0.001
Azerbaijan	58.0	2.65	3876.94	15.58	Yes	0.094
Bangladesh	34.0	5.73	1358.78	16.89	Yes	0.007
Belarus	90.0	3.54	4986.50	85.13	Yes	0.200
Belgium	84.0^a^	7.77	41,236.27	42.80	No	0.002
Bhutan	40.0^a^	4.93	2773.55	1.32	No	0.000
Bolivia	73.0	5.63	3104.96	9.94	Yes	0.175
Botswana	85.0	7.87	4808.41	44.71	Yes	15.998
Brazil	70.0^a^	6.90	8649.95	382.53	No	0.400
Bulgaria	64.0^a^	7.01	7469.03	12.57	No	0.049
Burundi	75.0	2.40	285.73	26.85	Yes	0.798
Cameroon	58.0	3.46	1374.51	30.58	No	2.389
Canada	78.8^a^	9.15	42,157.93	103.02	No	0.002
Chile	69.0	7.78	13,792.93	72.52	No	0.341
China	68.0^a^	3.14	8123.18	233.54	Yes	0.001
Colombia	50.8^a^	6.67	5805.61	20.91	Yes	0.247
Comoros	38.0	3.71	775.08	0.40	No	0.025
Costa Rica	53.0^a^	7.88	11,824.64	16.07	No	0.268
Croatia	65.0^a^	6.75	12,160.11	6.64	No	0.036
Cuba	87.0	3.46	7602.26	65.20	No	0.218
Czech Republic	75.0	7.82	18,491.94	35.22	No	0.032
Denmark	91.0^a^	9.20	53,549.70	19.33	No	0.001
Dominican Republic	69.0	6.67	6722.22	39.07	Yes	0.629
Ecuador	92.0	5.81	6018.53	5.22	Yes	0.201
Egypt	57.0	3.31	3477.85	16.34	No	0.011
Estonia	84.0^a^	7.85	17,727.49	5.96	No	0.007
Ethiopia	67.0	3.60	706.76	217.55	No	0.693
Fiji	87.0	5.64	5233.47	0.42	Yes	0.111
France	84.0^a^	7.92	36,854.97	178.40	No	0.269
Gabon	79.0	3.74	7179.34	6.79	No	2.425
Gambia	35.0	2.91	473.19	6.36	Yes	0.981
Georgia	42.0^a^	5.93	3865.79	17.53	Yes	0.323
Germany	85.0^a^	8.63	42,069.60	261.01	No	0.001
Ghana	45.0	6.75	1513.46	83.77	No	1.028
Greece	78.0^a^	7.23	17,930.16	21.86	No	0.002
Guatemala	65.0	5.92	4146.74	32.45	Yes	0.277
Guyana	69.0	6.25	4529.14	3.65	No	1.099
Haiti	59.0	4.02	739.60	53.48	No	1.383
Honduras	61.0	5.92	2361.16	23.26	No	0.230
Hungary	87.0^a^	6.72	12,814.95	9.82	No	0.001
India	77.0	7.81	1709.59	303.69	No	0.159
Indonesia	35.0	6.97	3570.29	66.62	No	0.237
Iran	38.0	2.34	5219.11	151.36	No	0.082
Ireland	85.0	9.15	63,861.92	12.94	No	0.130
Israel	74.0^a^	7.85	37,175.74	12.41	No	0.001
Italy	88.0^a^	7.98	30,674.84	171.93	No	0.215
Jamaica	79.0	7.39	4878.58	17.00	No	0.010
Japan	85.6^a^	7.99	38,900.57	142.03	No	0.000
Kazakhstan	74.0	3.06	7713.55	25.63	Yes	0.146
Kyrgyzstan	61.0	4.93	1077.04	21.39	Yes	0.140
Lesotho	72.0	6.59	1039.70	41.63	No	14.974
Liberia	33.0	5.31	455.37	20.58	Yes	0.932
Lithuania	88.0	7.47	14,879.68	2.09	Yes	0.101
Luxembourg	87.0^a^	8.81	10,0573.14	1.83	No	0.002
Madagascar	7.0	5.07	401.74	12.71	Yes	0.125
Malawi	70.0	5.55	300.31	221.91	No	5.527
Malaysia	95.0	6.54	9508.24	25.69	No	0.311
Malta	75.0	8.39	25,172.50	1.06	Yes	0.114
Mexico	69.2^a^	6.47	8208.56	150.54	No	0.172
Moldova	57.0^a^	6.01	1900.20	4.15	Yes	0.422
Mongolia	35.0	6.62	3694.08	9.44	No	0.017
Montenegro	76.0^a^	5.72	7023.54	1.55	Yes	0.080
Morocco	63.0	4.77	2832.43	11.45	No	0.062
Mozambique	61.0	4.02	382.07	193.77	Yes	6.244
Namibia	77.0	6.31	4414.98	83.94	No	9.275
Nepal	56.0	4.86	729.12	29.73	No	0.110
Netherlands	88.0^a^	8.80	45,669.81	58.55	No	0.135
Nicaragua	85.0	4.81	2151.38	26.96	Yes	0.145
Nigeria	34.0	4.50	2175.67	211.11	Yes	1.721
Niger	35.0	3.96	364.17	11.55	Yes	0.232
Panama	75.0	7.13	13,680.24	21.19	Yes	0.521
Papua New Guinea	81.0	6.03	2500.09	37.68	Yes	0.569
Paraguay	66.0	6.27	4077.74	8.04	Yes	0.283
Peru	53.7^a^	6.65	6049.23	23.91	No	0.220
Philippines	67.0	6.94	2951.07	16.40	No	0.054
Poland	57.0^a^	6.83	12,421.32	64.46	Yes	0.001
Portugal	70.0^a^	7.86	19,839.64	35.59	No	0.289
Romania	89.0	6.62	9519.88	48.73	Yes	0.081
Rwanda	87.0	3.07	702.84	125.85	No	1.846
Serbia	63.0^a^	6.57	5426.90	9.66	Yes	0.038
Sierra Leone	35.0	4.55	505.20	8.90	Yes	0.906
Singapore	69.0^a^	6.38	52,962.49	17.67	Yes	0.001
Slovakia	79.0	7.29	16,535.92	9.49	Yes	0.018
South Africa	86.0	7.41	5284.60	585.63	No	12.699
Spain	82.0^a^	8.30	26,639.74	182.42	No	0.301
Sri Lanka	47.0	6.48	3835.39	6.96	No	0.019
Sudan	39.0	2.37	2415.04	9.07	No	0.141
Suriname	62.0	6.77	5871.44	2.24	Yes	0.878
Sweden	90.0^a^	9.39	51,949.27	30.03	No	0.111
Switzerland	82.0^a^	9.09	79,890.52	27.74	No	0.189
Tajikistan	48.0	1.89	795.84	13.29	No	0.160
Tanzania	70.0	5.76	879.19	455.88	Yes	2.519
Thailand	91.0	4.92	5910.62	51.97	No	0.653
Togo	63.0	3.32	578.46	13.68	Yes	1.315
Tunisia	58.0	6.40	3688.65	7.35	No	0.025
Uganda	74.0	5.26	580.38	321.47	Yes	3.374
Ukraine	56.0	5.70	2185.73	126.77	Yes	0.533
United Kingdom	87.0^a^	8.36	40,341.41	178.82	No	0.158
United States	85.0^a^	7.98	57,638.16	615.71	Yes	0.004
Uzbekistan	51.0^a^	1.95	2110.65	22.51	Yes	0.138
Venezuela	54.3^a^	4.68	15,692.41	8.90	No	0.380
Vietnam	70.0	3.38	2214.39	67.52	Yes	0.270
Zambia	66.0	5.99	1269.57	148.84	No	7.233
Zimbabwe	75.0	3.05	1029.08	220.44	Yes	8.049

GDP, gross domestic product; PLHIV, people living with HIV; USD, US dollars.

a From independent sources.

Table 2 summarizes the linear regression models estimating the associations between our chosen independent variables and the primary outcome of interest, PLHIV who know their status. Multivariate linear regression analyses revealed that democracy index scores (β = 2.10, SE = 1.02, *p* = 0.04) and GDP per capita (in thousands; β = 0.34. SE = 0.11, *p* < 0.01) were positively associated with diagnosis rates among PLHIV, controlling for country‐level expenditure on HIV prevention, HIV criminalization laws and estimated HIV prevalence.

**Table 2:** Unstandardized coefficients and standard errors of OLS regression models estimating the percentage of PLHIV who know their status (n = 111)

	Percentage of PLHIV who know their status				
	Model 1	Model 2	Model 3	Model 4	Model 5
Constant	41.01 (4.94)***	48.31 (5.44)***	47.36 (5.40)***	47.83 (5.93)***	48.01 (5.88)***
Democracy index	4.40 (0.79)***	2.52 (1.02)*	2.37 (1.01)*	2.34 (1.02)*	2.10 (1.02)*
GDP per capita (current USD in thousands)	‐	0.30 (0.11)**	0.30 (0.10)**	0.30 (0.11)**	0.34 (0.11)**
HIV spending on prevention (US$ in millions)	‐	‐	0.03 (0.01)	0.03 (0.01)	0.02 (0.01)
Laws that criminalize HIV	‐	‐	‐	−0.61 (3.14)	−0.44 (3.11)
Estimated HIV prevalence	‐	‐	‐	‐	1.00 (0.58)
R^2^	0.221	0.274	0.300	0.300	0.319
Adjusted R^2^	0.213	0.261	0.280	0.273	0.287

GDP, gross domestic product; HIV, human immunodeficiency virus; PLHIV, people living with HIV; USD, US dollars.

*p < 0.05, **p < 0.01, ***p < 0.001.

Results of multivariate linear regression in Table 2 indicated that on bivariate analysis (Model 1), the democracy index score exhibited a strong positive association with the primary outcome variable, percentage of PLHIV who know their status. This positive association remained even after accounting for potential confounders that may be associated with both the democracy index score, and the proportion of PLHIV who know their status. This finding supports our hypothesis that given democratic political conditions, we would expect higher levels of PLHIV who know their status, by way of greater civil liberties and autonomy on the part of CSOs to collectivize and engage communities for HIV prevention efforts. However, further research is warranted to further substantiate this claim.

Results also indicated that GDP per capita was associated with higher levels of PLHIV who know their status within a country. This result is unsurprising as studies across different settings and among various key populations have found a positive association between an individual's socioeconomic status and voluntary HIV testing 37, 38, 39. Surprisingly, health expenditure on HIV prevention efforts, that would typically include HIV testing education and awareness campaigns or interventions, was not statistically significant. This is despite it being positively associated with the primary outcome at the bivariate level (β = 0.03, SE = 0.02, *p* = 0.03). However, its effect might have already been accounted for by the positive association between a country's GDP per capita and PLHIV who know their status, as a country's general wealth might also be proportionate to the absolute value of money spent on HIV prevention efforts.

Results also indicated that the existence of laws that criminalize HIV was not statistically significant with the proportion of PLHIV who know their status on bivariate analysis (β = −6.56, SE = 3.45, *p* = 0.06), but nonetheless indicated that the existence of such laws is associated with lower levels of PLHIV who know their status. The effect of the existence of HIV criminalization laws was largely accounted for in the multivariate model, and was not statistically significant, likely due to the model accounting for democratic political conditions, which exhibited higher measures in countries without the existence of such laws (t(109) = 3.21, *p* < 0.01). Finally, results also indicated that HIV prevalence did not have a statistically significant association with PLHIV who know their status, at both bivariate and multivariate levels of analyses.

We are mindful of the study's limitations. First, as broad country‐level indicators were used, the results of this study are subject to the ecological fallacy. For example, GDP per capita may not necessarily translate to individual‐level socioeconomic status, which forms part of the specific mechanism or pathway that might lead an individual to test for HIV. However, we believe that this potential bias does not detract from the strength of the assertions that this study attempts to put forth, as country‐level data collected in this study is not meant to be extrapolated to individual exposure measures, but rather, remains as a structural exposure that may apply to a broader population of individuals 40.

Second, due to the lack of standardized, available data on several measures of interest at the country level, proxy data were collected instead which could have biased the findings of this study. First, the democracy index was a composite, average score that was derived from five component measures of “electoral process and pluralism,” “civil liberties,” “functioning of government,” “political participation” and “political culture.” Thus, the composite score or any of its component measures may not have comprehensively captured measures that would specifically impact the ability of CSOs to organize and deliver HIV prevention services in a given setting. Second, as a uniform measure of HIV stigma data was not available for most countries, a proxy variable of the existence of HIV criminalization laws was used instead, which may or may not reflect levels of HIV stigma that individuals face and thus may prevent them from getting tested.

Third, several important confounders may not have been adequately accounted for in our analysis. Although educational attainment was identified as a potential confounder in the present analysis, country‐level measures of educational attainment were inadequate or inappropriate for use. For example, available country‐level data such as the United National Development Programme Education Index is calculated from a country's mean years of schooling divided by the expected years of schooling, which accounts for intra‐country, but not inter‐country differences in educational attainment or literacy. Past studies that find educational attainment as being positively associated with voluntary HIV testing have only comparatively done so at the level of the individual in both general and key populations that are affected by HIV 41, 42, 43.

Finally, the lack of time‐series or panel data that tracked measures of our outcome and exposure variables over multiple years limits the current analysis to claiming an association between measures of democracy and PLHIV who know their status, rather than causation. However, we restricted our analysis to a single year as data points of the outcome variable that were derived from other scientific sources or national reports, as well as other important covariates to the analysis, including data on HIV criminalization and estimates on national spending on HIV prevention, were only available for a single year and without repeated estimates. In the interest of greater analytic rigor, and as country‐level reporting mechanisms and methods for measuring the 90‐90‐90 targets improve in tandem with on‐the‐ground programme implementation 44, a prospective longitudinal dataset comprising variables in the present analysis may be constructed to strengthen and validate the findings of this study.

## 4 Conclusions

The present study draws on a global analysis to illustrate that a higher measure of democracy through the democracy index was associated with a higher proportion of PLHIV who know their status, and this relationship held even after adjusting or controlling for potential confounders such as country‐level GDP per capita, HIV prevalence, the existence of HIV criminalization laws and healthcare expenditure on HIV prevention efforts. We hypothesize that the mechanism through which this takes effect would be through the increased capacity and opportunities for CSOs to organize, articulate communities’ healthcare needs and engage in healthcare delivery for their own communities. This study thus contributes to the literature in this respect, and hopes to provide additional evidence that would emphasize the role of politico‐legal structures as barriers or facilitators to HIV prevention across the globe. However, further research is warranted to further substantiate this claim.

We recommend that countries respond to the global HIV/AIDS epidemic through structural interventions that will facilitate the work of CSOs. This includes the removal of laws that criminalize PLHIV or key populations, as we have seen how oppressive regimes and laws drive key populations, PLHIV, and others who are impacted by HIV further away from meaningful engagement in care 45, 46, 47. Other interventions that have been found to positively impact community‐based programmes include greater state‐civil society engagement that include the provision of tangible and intangible resources, funding for community‐based grassroots initiatives and capacity building for CSOs 48, 49. Ultimately, we do recognize that there may be nuances in state‐civil society interactions that may restrict or facilitate efforts by CSOs which may not be reflected in the current measure of democracy, and thus further research on such dynamics is warranted. In spite of the limitations of this study, we believe that this analysis presents evidence to underscore the importance of structural conditions in allowing CSOs to engage in the HIV/AIDS response of their respective communities and countries.

## Competing interests

The authors declare no competing interests for this study.

## Authors’ contributions

RKJT conceptualized the study and collected, cleaned and analysed the data. RKJT and CSW wrote and reviewed the paper.
